# Presentations and mechanisms of CNS disorders related to COVID-19

**DOI:** 10.1212/NXI.0000000000000923

**Published:** 2020-12-09

**Authors:** Marta Bodro, Yaroslau Compta, Raquel Sánchez-Valle

**Affiliations:** From the Infectious Diseases Department (M.B.), Hospital Clínic de Barcelona, Catalonia; Institut d'Investigacions Biomèdiques August Pi i Sunyer (IDIBAPS) (M.B., Y.C., R.S.-V.), Barcelona; Department of Medicine (M.B., Y.C., R.S.-V.), Universitat de Barcelona; Neurology Department (Y.C., R.S.-V.), Hospital Clínic de Barcelona; and Institut de Neurociències (Y.C., R.S.-V.), Maria de Maeztu Excellence Center, Universitat de Barcelona, Catalonia, Spain.

## Abstract

Severe acute respiratory syndrome coronavirus 2 (SARS-CoV-2) is the cause of the coronavirus disease 2019 (COVID-19) pandemic. In addition to severe respiratory symptoms, there are a growing number of reports showing a wide range of CNS complications in patients with COVID-19. Here, we review the literature on these complications, ranging from nonspecific symptoms to necrotizing encephalopathies, encephalitis, myelitis, encephalomyelitis, endotheliitis, and stroke. We postulate that there are several different mechanisms involved in COVID-19–associated CNS dysfunction, particularly activation of inflammatory and thrombotic pathways and, in a few patients, a direct viral effect on the endothelium and the parenchyma. Last, critically ill patients frequently present with protracted cognitive dysfunction in the setting of septic encephalopathy likely due to multifactorial mechanisms. Further studies are needed to clarify the relative contribution of each of these mechanisms, but available data suggest that CNS complications in COVID-19 are rare and probably not directly caused by the virus.

Severe acute respiratory syndrome coronavirus 2 (SARS-CoV-2), the cause of the coronavirus disease 2019 (COVID-19) pandemic, produces respiratory symptoms similar to those of SARS-CoV and Middle East respiratory syndrome coronavirus. The most dreadful complication is respiratory distress, with many patients requiring intensive care management.^1^ The viral infection activates inflammatory and prothrombotic pathways, which might be relevant for the pathogenesis of systemic and CNS symptoms.

Coronaviruses can also invade the CNS. In the case of SARS-CoV-2, trans-synaptic propagation from initial involvement of the olfactory epithelium and nerve is 1 proposed route. Accordingly, hyposmia and dysgeusia are common in COVID-19 and some studies suggest may associate with good prognosis.^2–4^ Alternatively, the virus might reach the CNS via the blood.^5^

The frequency and types of neurologic complications in patients with COVID-19 are being elucidated. Initial studies showed that patients with mild cases of COVID-19 present with headache or dizziness, whereas those with severe disease can also develop an encephalopathy with agitation, confusion, impaired consciousness, seizures, and signs of corticospinal tract dysfunction.^6^ Ischemic stroke also occurs in COVID-19 and might be associated with a viral-related systemic prothrombotic state.^7^ Isolated cases with acute COVID-19–associated encephalitis have been reported, but demonstration of the presence of SARS-CoV-2 in the brain or CSF has been inconsistent.^8,9^

In Spain, there have been at least 257,000 patients affected with COVID-19. The Albacete COVID-19 registry included 841 cases with neurologic symptoms, including 14 cases of ischemic or hemorrhagic stroke, totaling less than 2% of the series. Only 1 patient with encephalitis, 1 with optic neuritis, and 1 compatible with posterior reversible encephalopathy syndrome (likely related to hypertension) were recorded.^10^

The aims of the present article are to review CNS complications associated with SARS-CoV-2 infection and analyze potential mechanisms of CNS damage.

## Literature search strategy

We search PubMed for articles with clinical information on patients with CNS disorders related to SARS-CoV-2 infection. We excluded reports where CNS dysfunction was drug induced or could be explained by a nonspecific response to systemic infection, particularly in patients with preexisting structural lesions in the CNS. We also excluded (1) case reports on isolated cranial nerve (including hyposmia) or peripheral nervous system involvement (for this topic, see the recent review by Dalakas,^11^ (2) reviews and recommendations about treatment of preexisting neurologic disorders in the setting of COVID-19, and (3) studies on certain treatments used for neurologic diseases that could modify the risk or course of COVID-19.

### Overview of CNS complications of SARS-CoV-2

Observational series have reported the presence of CNS symptoms in 31%^6^ to 69%^2^ of patients with severe COVID-19 vs 21% in patients with nonsevere COVID-19 (severity being established according to respiratory status). Dizziness (17%), headache (13%), impaired level of consciousness (8%), acute stroke (3%), ataxia (<1.0%), and seizures (<1.0%) were the main symptoms or syndromes reported in hospitalized patients.^6^ Agitation (69%), confusion (65%), signs of corticospinal tract dysfunction (67%), and impairment of executive functions (36%) were frequent symptoms in an observational series of 64 consecutive patients admitted to 2 intensive care units (ICUs) due to acute respiratory distress syndrome caused by COVID-19.^2^ In this series, at the time of hospital discharge, 33% of patients had a dysexecutive syndrome consisting of inattention, disorientation, and poorly organized responses to commands.^2^

In patients with COVID-19 admitted in the ICU with neurologic symptoms, the main MRI abnormalities include cortical FLAIR signal abnormalities^12^ and silent ischemic strokes.^2^ In one of these series,^12^ only 7 of the 58 patients (12%) underwent real-time PCR for SARS-CoV-2 in CSF, and all were negative. In the other series the patients who underwent EEG, only nonspecific changes were detected.^2^ A recent single case report of a patient with COVID-19 who needed extracorporeal membrane oxygenation (ECMO) had documented brain microbleeds as seen in other critical illness cases requiring ECMO and previously associated with influenza virus. The authors suggested an underlying endotheliopathy.^13^

A retrospective multicenter case series from France showed that stroke, leptomeningeal enhancement, and encephalitis were the 3 most frequent neuroimaging findings among 64 patients with COVID-19.^14^ Confusion, impaired consciousness, agitation, corticospinal tract signs, and headache were the most common clinical features. Encephalitis occurred more frequently in younger patients, and agitation was associated with leptomeningeal enhancement.^14^

Finally, in a case series from the United Kingdom that included 43 patients with definitive (n = 29), probable (8), and possible (6) COVID-19, 5 categories of neurologic involvement emerged, with 3 specifically affecting the CNS: encephalopathies (n = 10); inflammatory CNS syndromes (n = 12) including encephalitis, acute disseminated encephalomyelitis (ADEM), and myelitis; and ischemic strokes (n = 8).^15^

Other than these series, well-characterized case reports with predominant CNS involvement other than stroke (reviewed separately) are scarce and are summarized in the table. In short, these include 14 cases, 9 of them (75%) were women, with age ranging from 24 to 69 years. Three were diagnosed with encephalitis, 4 with acute necrotizing encephalopathy, 2 with a clinicoradiologic picture compatible with endotheliitis (without pathologic confirmation), 2 with toxic-metabolic or vitamin deficiency–like encephalopathy, 2 with ADEM, and 1 with necrotizing myelitis.^8,9,16–25^ Of these 14 cases, only 3 had positive SARS-CoV-2 PCR in CSF (2 with negative nasopharyngeal swabs). In the remaining 10 cases, SARS-CoV-2 infection was demonstrated with nasopharyngeal PCR.

**Table:**
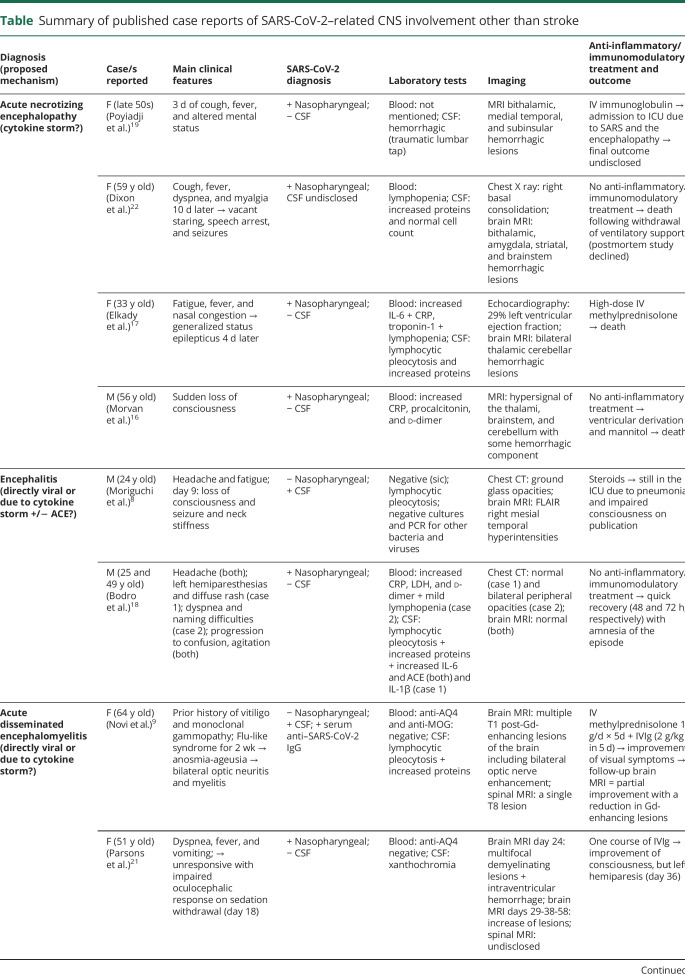
Summary of published case reports of SARS-CoV-2–related CNS involvement other than stroke

### Acute necrotizing encephalopathy (n = 4)

The 4 cases reported so far with acute necrotizing encephalopathy resemble those seen after other viral infections, mainly influenza. All these cases had negative PCR for SARS-CoV-2 in CSF.^19,22,25,26^ Systemic inflammatory response syndrome (SIRS) and cytokine storm are also thought to underlie this condition. One of the patients also developed myocarditis.^16,17^ Two of the patients did not respond to treatment and died.^16^ An additional case without data on sex and age but with positive PCR in CSF and improvement with IV immunoglobulins (IVIgs) and plasma exchange has been reported.^26^

### Encephalitis (n = 3)

The 3 reported cases with encephalitis differ in terms of clinical severity and CSF and MRI features.^8,18^ Thus, although all 3 showed CSF lymphocytic pleocytosis with increased protein concentration, 1 was severely ill, had positive PCR for SARS-CoV-2 in CSF, and showed structural lesions (mostly in the mesial right temporal lobe) on brain MRI.^8^ By contrast, the other 2 patients had rapid recovery, negative CSF PCR for SARS-CoV-2, and normal MRI.^18^ Of interest, these 2 patients were the first reported cases in whom CSF levels of interleukins (ILs) and angiotensin-converting enzyme (ACE) were measured, showing increased levels of IL-1β (in both), IL-6 (in 1), and ACE (both).^18^ Accordingly, encephalitis of the former case might be related to direct CNS infection,^8^ whereas in the other 2, it could be linked to cytokine storm and endothelial inflammation as suggested by increased ILs and ACE levels in CSF.^18^

### ADEM (n = 2) and acute necrotizing myelitis (n = 1)

ADEM is a clinicoradiologic condition that requires a multifocal CNS event of presumed inflammatory demyelinating cause, with an encephalopathic picture that cannot be explained by fever or other causes. It requires the presence on MRI of diffuse, poorly demarcated, large (>1–2 cm) lesions mostly involving the white matter, rarely with T1 hypointense white matter lesions.^27^ Deep gray nuclei (thalami and basal ganglia) lesions can be seen, without additional clinical and MRI findings in the ensuing 3 months. ADEM is not associated with aquaporin 4 (AQP4) antibodies, but up to 60% of patients have myelin oligodendrocyte glycoprotein (MOG) antibodies.

To the best of our knowledge, there are 2 single case reports of ADEM associated with COVID-19.^9,21^ One had positive SARS-CoV-2 PCR in CSF and a clinical picture that resembled neuromyelitis optica and was treated with IV methylprednisolone and IVIg.^10^ The other patient had a negative PCR in CSF and developed decreased level of consciousness and impaired oculocephalic response after a stay in the ICU due to respiratory distress. Despite the diagnosis of ADEM, findings of spinal MRI were not reported. This patient was treated with IVIg.^21^ Both patients had partial clinical and radiologic responses. A single case report of a patient with longitudinal extensive transverse necrotizing myelitis who again had a negative PCR for SARS-CoV-2 in CSF, was also negative for AQP4 and anti-MOG antibodies, and responded to methylprednisolone and plasmapheresis.^25^

Overall, in these 3 cases, the different evidence of the infectious status makes it difficult to infer the underlying pathophysiology. Although in 2 of the patients, the CSF PCR can potentially be considered a false-negative result, the clinical and radiologic features of these cases are suggestive of an inflammatory postinfectious process.

### Endotheliitis-like involvement (n = 2)

The 2 reported cases of endotheliitis-like involvement include 1 patient with focal neurologic signs and the other with impaired level of consciousness. Both had marked perivascular enhancement on MRI, were negative for SARS-CoV-2 PCR in CSF, and had rapid clinical and radiologic recovery after treatment with IV methylprednisolone and plasma exchange, supporting the notion of an inflammatory mechanism.^20,23^ Although no pathologic studies were available, the MRI findings led to the diagnosis of endotheliitis. The inflammatory endothelial reaction might be related to cytokine storm or the ACE pathway, but neither of these mechanisms was studied in blood or CSF.

More recently, Pugin et al.^28^ reported 5 cases of COVID-19–related encephalopathy responsive to corticosteroids that the authors suggested were caused by endotheliitis based on the MR angiography features.

### Toxic/Wernicke-like encephalopathy (n = 2)

Two patients were identified at a single institution with COVID-19 who had developed clinical and brain MRI features that resembled Wernicke encephalopathy, prompting supplementation of vitamins despite having normal blood levels.^24^ The patients showed partial improvement. The authors considered the symptoms as related to SARS-CoV-2, either by direct viral involvement or secondary to the systemic inflammatory response to the virus.

### Stroke

In a series of 388 patients, thromboembolic events occurred in 28 patients (8%) including 9 cases with ischemic stroke (2.5%).^29^ Likewise, in a study of ischemic events in critically ill patients with COVID-19, 28 of 184 cases had thrombotic events (15%), including 3 with stroke.^30^ In another series of 1,683 COVID-19 cases, 23 (1.4%) developed cerebrovascular disease.^31^ Cerebral and chest CT scans were performed in all cases and MRI in 6, with histologic samples available for 6/23 cases (2 brain biopsies and 4 arterial thrombi). Cerebral ischemia occurred in 17 cases (74%), including 2 cases with arterial dissection (9%), whereas intracerebral hemorrhage happened in 5 (22%), and 1 (4%) had posterior reversible encephalopathy. Ischemic strokes were frequent in the vertebrobasilar territory. In the hemorrhagic group, subarachnoid hemorrhage, parieto-occipital leukoencephalopathy, microbleeds, and single or multiple focal hematomas were characteristic. Brain biopsies showed thrombotic microangiopathy and endothelial injury, with no evidence of vasculitis or necrotizing encephalitis. Outcome was poor, 8 died, and 17 had a median Rankin scale score of 4–6 at hospital discharge.^31^

In another study, Al Saiegh et al. reported 2 patients with cerebrovascular disease in the setting of COVID-19. One was a 31-year-old man with a Hunt & Hess grade 3 subarachnoid hemorrhage and the other a 62-year-old woman with ischemic stroke with massive hemorrhagic transformation.^32^ Oxley et al. reported 5 patients with COVID-19 younger than 50 years with large vessel ischemic stroke,^33^ and Gonzalez-Pinto et al.^34^ described a 36-year-old man with malignant ischemic stroke of the middle cerebral artery and a free-floating thrombus in the ascending aorta. Overall, these reports suggest that stroke is more frequent than expected in patients with COVID-19 younger than 50 years.

### Other CNS involvement

There are other case reports difficult to classify. For example, Méndez-Guerrero et al. described a 50-year-old man presenting with a hypokinetic syndrome, hyposmia, opsoclonus, and myoclonus after a severe COVID-19 infection with positive nasopharyngeal but negative CSF PCR. The MRI was normal, but there was impaired presynaptic dopamine imaging. The patient spontaneously recovered.^35^ In another report, Rabano-Suarez and colleagues^33,36^ described 3 cases (2 men, 63 and 76 years and a woman of 88 years) with COVID-19 who had generalized myoclonus with normal CSF, imaging (MRI or CT), and EEG. They slowly improved with either corticosteroids or plasma exchange, leading the authors to conclude that the myoclonus was postinfectious and immune mediated.

## Mechanisms of CNS damage associated with SARS-CoV-2

### Direct viral injury

Like other well-recognized neuroinvasive human viruses, coronavirus might invade the CNS, via trans-synaptic propagation from the olfactory epithelium or through systemic circulation, entering the CNS using the endothelial ACE2 receptors expressed in brain vessels, or crossing a leaky blood-brain barrier affected by systemically produced cytokines (figure).^37^

**Figure:**
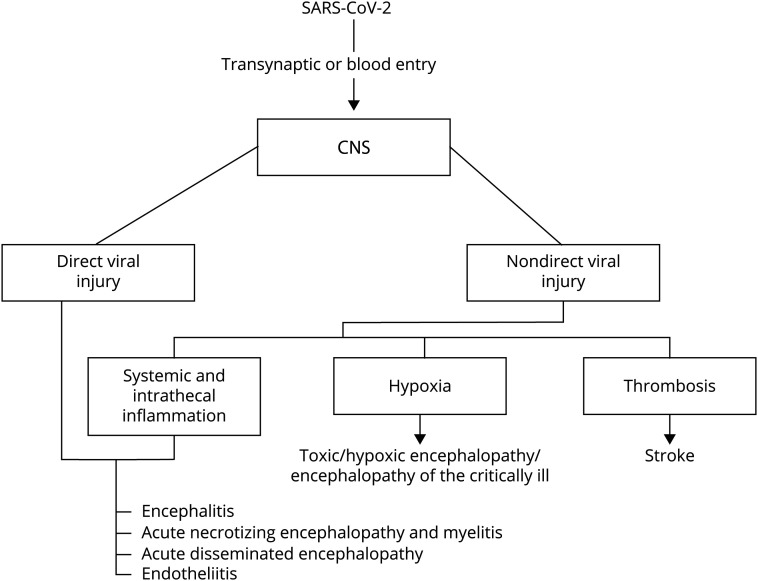
Proposed CNS entry routes, mechanisms and their respective associated clinical pictures

Therefore, SARS-CoV-2 might reach the CNS by different routes and induce short-term illnesses, such as viral encephalitis. On the other hand, SARS-CoV-2 might persist in resident cells of the CNS and be involved in long-term neurologic sequelae in genetically or otherwise predisposed individuals.^37^

There are case reports of the detection of human coronavirus OC43 RNA in brain biopsies by metagenomic sequencing in immunosuppressed children with encephalitis.^38,39^ Moreover, SARS-CoV-1 has also been detected at autopsy in brain tissue of patients with SARS.^40–42^ However, as previously indicated, there are limited data suggesting the presence of SARS-CoV-2 in the CNS of patients with COVID-19. In an autopsy series of patients with COVID-19 without neurologic symptoms, SARS-CoV-2 was detected in the brain of 8 (38%) of 21 patients with low levels of SARS-CoV-2 copies per cell.^43^ In another autopsy study of 10 patients,^44^ no signs of encephalitis or CNS vasculitis were found, and the PCR in CSF was negative.

Only 2 of the 11 patients who underwent PCR testing in CSF (table) were positive (1 case of encephalitis and 1 ADEM), suggesting that the virus can directly invade the nervous system in some cases.^8,9^

#### Neuronal pathway

Some human coronaviruses such as HCO-OC43, HCOV-229E, and SARS-CoV-1 can be considered neurotropic viruses due to their capacity to invade the CNS via the neuronal pathway.^5,37,45^ Viruses can migrate by infecting sensory or motor nerve endings, achieving retrograde or anterograde neuronal transport through kinesins, dynein, and motor proteins.^46^ Experimental intranasal coronavirus infection in susceptible mice shows that once the virus has invaded the CNS through the olfactory route, it can disseminate to several regions of the brain and brainstem before eventually reaching the spinal cord.^45,47^

#### Blood circulation pathway

Viruses can enter the CNS without infecting neurons. Some viruses, such as HIV, infect leukocytes and may infiltrate the brain parenchyma. This Trojan horse mechanism is facilitated by the fact that the infected cells are naturally able to cross the blood-brain barrier.^48^ Alternatively, other viruses such as Japanese encephalitis virus are released into the blood and increase the permeability of the blood-brain barrier through increased production of proinflammatory cytokines that facilitates entry into the CNS.^49^ This mechanism may be plausible for SARS-CoV-2 due to the severe systemic inflammation with elevated cytokine levels that can constitute a link between direct CNS infection and CNS damage due to systemic and intrathecal cytokine storm.^50^ Alternatively, expression of ACE2 receptors in the endothelium of brain blood vessels provides an additional entry route for SARS-CoV-2 that can be linked with endotheliitis and inflammatory injury of the CNS (see below).^51^ Further studies analyzing how SARS-CoV-2 invades the CNS are warranted.

### Inflammatory-mediated injury

#### Cytokine storm

COVID-19 has caused a large number of fatalities, most due to multiple organ failure secondary to the virus-induced SIRS or SIRS-like immune disorders.^52^ Accumulating evidence suggests that patients with severe COVID-19 might have a cytokine storm syndrome characterized by increased IL-1, IL-2, IL-6, granulocyte-colony stimulating factor, interferon-γ inducible protein 10, macrophage inflammatory protein 1α, and tumor necrosis factor (TNF)-α.^53^ Lung tissue from patients with SARS related to coronavirus showed hemophagocytosis, which is a central pathologic feature of cytokine storm.^54^ Patients with SARS also exhibited high levels of interferon-γ and IL-18, which are particularly important in the cytokine storm syndrome.^55^ Thus, the host's immune response and development of inflammation in the lung likely plays an important role in COVID-19.^56^

Li et al.^57^ found that coronavirus neurovirulence correlates with the ability of the virus to induce proinflammatory cytokine (IL-12 p40, TNFα, IL-6, IL-15, and IL-1β) signaling from astrocytes and microglia in a mouse model. Other investigators also showed that primary glial cell cultures exposed to coronavirus secrete the proinflammatory cytokines IL-6, IL-12, IL-15, and TNFα.^58^

In cases of acute necrotizing encephalopathy, acute necrotizing myelopathy, encephalitis, and ADEM with negative PCR in CSF, cytokine storm is the most probable mechanism of CNS dysfunction. A recent single autopsy case report described a combination of hemorrhagic white matter lesions with areas of necrosis and organizing microscopic infarcts, along with perivascular ADEM-like alterations, suggesting a combination of vascular and inflammatory injury.^59^

The hyperinflammatory response that leads to SIRS can manifest with high levels of C-reactive protein, coagulopathy (elevated d-dimer levels, low platelet count, and fibrinogen levels), tissue damage (elevated LDH and alanine aminotransferase and aspartate aminotransferase levels), macrophage/hepatocyte activation (elevated ferritin levels), and cytopenias (thrombocytopenia and lymphopenia).^56^ Therefore, early anti-inflammatory intervention such as anticytokine therapies could reduce the risk of injury in the nervous system.^60^ Glucocorticoids remain the first treatment option, and clinical trials are urgently needed to test their efficacy and identify optimal dosing, especially given the clear advantage of glucocorticoids in worldwide availability and cost.^61^

#### Endotheliitis

The binding of SARS-CoV-2 to the ACE2 receptor may cause or worsen high blood pressure, increasing the risk of cerebral hemorrhage. Moreover, the interaction of the SARS-CoV-2 spike protein with ACE2 receptors expressed in the capillary endothelium of brain blood vessels may lead to injury of the blood-brain barrier. This could explain the increased risk of ischemic stroke (see below) accompanied by perivascular inflammation suggestive of endotheliitis.

### Thrombotic injury

Increased levels of circulating prothrombotic factors have been reported in patients with severe COVID-19, reflecting a hypercoagulable state.^7,62^ Proinflammatory cytokines can induce endothelial and mononuclear cell activation with expression of tissue factor leading to activation of the coagulation cascade and thrombin generation. Circulation of free thrombin can activate platelets and lead to thrombosis. It has been postulated that the binding of SARS-CoV-2 to the ACE2 receptor causes an imbalance of the renin angiotensin system favoring the ACE1-angiotensin axis and contributing to endothelial dysfunction, organ damage, and clot formation.^63^

### Hypoxia-mediated injury and encephalopathy of the critically ill

The diffuse alveolar and interstitial inflammatory edema in COVID-19 leads to impairment of alveolar gas exchange and CNS hypoxia. Anerobic metabolism in the mitochondria of brain cells results in acidosis, vasodilatation, increased interstitial edema, obstruction of cerebral blood flow, intracranial hypertension, and coma.

Between 5%^1,2^ and 14%^3^ of patients with COVID-19 require admission to the ICU. A high number of patients in the ICU develop symptoms of acute cerebral dysfunction described as delirium, acute confusional state, acute brain failure, organic brain syndrome, acute organic reaction, cerebral insufficiency, and ICU psychosis and grouped under the term “encephalopathy of the critically ill” or “critical illness brain syndrome.” This encephalopathy is an independent predictor of mortality and is associated with long-term cognitive dysfunction.^64^ The mechanisms underlying this encephalopathy are heterogeneous and multifactorial, including direct brain injury, respiratory, heart, renal or liver failure, sepsis, endocrine or electrolyte imbalance, and pharmacologic agents.^65^

In a series of 18 patients with COVID-19 who died in the ICU and underwent autopsy, acute hypoxic ischemic damage was identified in all. Nevertheless, the clinical description is very limited, and none of the cases underwent MRI, EEG, or CSF examination.

## Conclusions

CNS involvement in COVID-19 appears to be protean both in its clinical presentations and underlying mechanisms. Direct viral infection of the CNS has been demonstrated in a few cases. In most patients with nonsevere forms of the disease (according to the respiratory status), inflammatory or cerebrovascular mechanisms appear to be the predominant CNS complications. In this context, the involved mechanisms include inflammatory (cytokine storm), prothrombotic, and endothelial (via ACE2) pathways. In critically ill patients with COVID-19, additional mechanisms include those typically related to encephalopathy of the critically ill, such as hypoxia, systemic disturbances, and drugs, among others.

Nevertheless, the number of patients with specific syndromes (encephalitis, ADEM, myelitis, and endotheliitis) is small, suggesting that the frequency of these complications is rare, making difficult an accurate classification. There are limited data to determine whether hypoxemic or post-ICU or septic encephalopathy is more frequent or severe in COVID-19 than in other respiratory infections that are similarly severe. Moreover, there is a need for more reliable and standardized biomarkers for infection of the CNS (PCR and antibodies against SARS-CoV-2 in CSF) and for the involvement of endothelial, inflammatory, and prothrombotic pathways within the CNS.

Last but not least, treatment strategies for these potential pathophysiologic pathways are limited. The usefulness of immunomodulatory treatments (corticosteroids and plasma exchange), anti-IL antibodies (tocilizumab and anakinra), full-dose anticoagulants, and others needs to be investigated and established.

In summary, both parenchymal (encephalitis, myelitis, or combination of both) and vascular (endotheliitis) CNS inflammatory responses along with an increased risk of hemorrhagic and ischemic stroke have been described as complications of COVID-19. However, severe CNS complications associated with COVID-19 are rare and probably not directly caused by the virus in most cases. Severe cases of COVID-19 frequently manifest as a syndrome that overlaps with encephalopathy of the critically ill, whereas fewer cases (at least to date) present with specific CNS syndromes. Further studies are needed to clarify the pathogenesis and long-term prognosis of CNS involvement in patients with COVID-19.

## Glossary

ACE: angiotensin-converting enzyme
ADEM: acute disseminated encephalomyelitis
AQP4: aquaporin 4
COVID-19: coronavirus disease 2019
ECMO: extracorporeal membrane oxygenation
ICU: intensive care unit
IL: interleukin
IVIg: IV immunoglobulin
MOG: myelin oligodendrocyte glycoprotein
SARS-CoV-2: severe acute respiratory syndrome coronavirus 2
SIRS: systemic inflammatory response syndrome
TNF: tumor necrosis factor
